# Transcriptomic analysis of bone marrow adipose tissue reveals enrichment in lipid transport in response to a Western diet

**DOI:** 10.1210/endocr/bqag092

**Published:** 2026-08-14

**Authors:** Nicko Widjaja, Niki Jalava, Leena Strauss, Kirsi A Virtanen, Kaisa K Ivaska

**Affiliations:** Institute of Biomedicine, University of Turku, Turku 20520, Finland; Institute of Biomedicine, University of Turku, Turku 20520, Finland; Institute of Biomedicine, University of Turku, Turku 20520, Finland; Turku PET Centre, University of Turku, Turku 20520, Finland; Turku PET Centre, Turku University Hospital, Turku 20520, Finland; Institute of Biomedicine, University of Turku, Turku 20520, Finland

**Keywords:** bone marrow adipose tissue, white adipose tissue, high-fat diet, transcriptomics, lipid metabolism

## Abstract

Bone marrow adipose tissue (BMAT) is now recognized as a functional fat depot distinct from extramedullary white and brown adipose tissues (WAT and BAT, respectively). BMAT expands in metabolic disorders such as obesity. However, the mechanism by which BMAT expands is less clear. Here, we present a comparative analysis of the transcriptomes of BMAT, WAT, and BAT from 20-week-old Sprague-Dawley rats fed a chow diet (12% kJ fat) using bulk RNA sequencing (RNA-seq). Gene-set enrichment analysis revealed overrepresentation of transcripts related to cellular proliferation and the population of adipoprogenitor cells in BMAT. To further study BMAT expansion, we introduced a 12-week dietary intervention of a Western diet (42% kJ fat). We first characterized obesity-related phenotypes such as adipocyte hypertrophy. Then, we compared the transcriptomes of femoral and tibial BMAT or gonadal WAT to the chow group. Western diet significantly increased body weight (*P* = .02), whole-body fat percentage (*P* = .009), adipocyte size in humeral BMAT (*P* = .02), and gonadal WAT (*P* = .005) compared with the chow group. BMAT had a more robust transcriptomic response to the Western diet than WAT. The transcriptome of BMAT was enriched in pathways related to lipid transport. In WAT, this enrichment was more related to lipid biosynthesis and modification. In summary, our data may suggest differences in the developmental maturity of BMAT relative to WAT and BAT in the chow group. A Western diet induces differential responses in the transcriptomes of BMAT and WAT related to lipid homeostasis. In BMAT, enrichment in the lipid transport pathways on the Western diet may suggest a role for BMAT in nutrient sensing and lipid uptake.

Bone marrow adipose tissue (BMAT) fills up to 70% of the bone marrow cavity in humans, comprising about 10% of our total fat mass (1-3). BMAT is enriched in the long bones and is increased with aging in both the axial and appendicular skeleton (2, 4). BMAT is now considered a fat depot distinct from white and brown adipose tissues (WAT and BAT, respectively) (5). We and others have shown that BMAT is a metabolically active fat depot (6-8). BMAT may locally regulate hematopoiesis and bone remodeling within the local bone marrow microenvironment (9-11) or contribute to systemic metabolism via secreted factors (1, 8, 12). The gene expression profile in BMAT differs from that of WAT and BAT in mice (13) and in humans (8, 12), although it is less clear what these collective differences convey functionally. Further, the lipid metabolism in human BMAT appears to be different from WAT, with profound alterations in the lipolytic pathway (14).

Bone marrow is a complex tissue containing multiple cell types and extensive spatial heterogeneity (15). Various stromal, nonadipocyte cells such as adipocyte precursor cells and immune cells reside closely around or adjacent to bone marrow adipocytes (BMAds) (16-18), also creating challenges for the isolation of BMAds from bone marrow. In mice, BMAds originate from preadipocyte-like perivascular progenitors (19) and are mostly associated with sinusoidal vasculature (16). Lineage tracing experiments in rodents have revealed 2 distinct stem cell-like adipogenic precursor populations, namely the adipoprogenitor cells (APCs) and fate-committed preadipocytes, which are identified through different sets of enriched transcripts (20, 21). However, further characterization of which population predominates at specific time points in bone marrow or their relative abundance in different adipose tissue depots is less well known.

BMAT is responsive to changes in metabolic status, such as during starvation or high-calorie intake, and the amount of BMAT fluctuates in most metabolic disorders in site-, age-, and species-specific manners (4, 21-23). However, due to the challenges in accessing viable adipocytes from BMAT, little is known about how BMAds respond to metabolic stimuli in comparison to the classical white adipocytes at the cellular level. Further, the molecular mechanisms for BMAT expansion upon dietary challenge are mostly unknown, and the limited data on nutrient-sensing pathways in BMAT come from targeted analysis of mouse models (13, 22, 24). We recently showed that BMAds expand in size in rats fed a high-fat, high-cholesterol Western diet for 24 weeks (6). However, the molecular mechanisms driving the hypertrophy of BMAds are poorly understood. It is unclear whether BMAds respond to diet similarly to classical white adipocytes (ie, through hypertrophy, hyperplasia, and inflammation) (25). The expansion of BMAds in rats upon a Western diet was relatively stable and was not significantly affected by moderate lifestyle interventions such as a switch back to standard diet and running exercise (6). Therefore, this study aimed to investigate the early changes in the transcriptomes of BMAT and WAT in response to a Western diet at a shorter intervention period of 12 weeks.

We hypothesized that the physiological function of BMAT is impaired upon metabolic challenge, and the response to metabolic challenge is reflected in the transcriptomic landscape of BMAds. Using bulk RNA-seq, here we report the transcriptomic characteristics enriched in BMAT isolated from femurs and tibiae of rats, in comparison to perigonadal and interscapular adipose tissues representing WAT and BAT, respectively. Next, we asked if the response to dietary challenge is different in BMAT and WAT. We used a 12-week intervention of a high-fat, high-cholesterol Western diet and compared the diet-induced transcriptomic responses in BMAT and WAT. Finally, we uncovered biological pathways through targeted analysis of adipose tissue transcriptomes, highlighting the differential response of BMAT and WAT to the dietary challenge.

## Materials and methods

### Animal experimentation

Eight-week-old male Sprague-Dawley rats (n = 30) were purchased from the Central Animal Laboratory of the University of Turku, Finland, and allocated into 2 intervention groups of similar body weight at baseline (both groups averaged 286 g; *P* > .99). Animals were fed with either a standard chow diet (n = 15; Envigo, USA; 0% cholesterol, 12.6 MJ/kg: 12% from fat, 22% from protein, 66% from carbohydrates), or a Western diet (n = 15, ssniff Spezialdiäten GmbH, Germany; 1.25% added cholesterol, 19.1 MJ/kg: 42% from fat, 15% from protein, 43% from carbohydrates) for 12 weeks. Rats were housed 3 to a cage under standard conditions of a 12-h light/dark cycle, 21 °C, and 55% humidity. Food and water were provided ad libitum. Food consumption was recorded by measuring the weight difference between the added and remaining food pellets 3 times a week. Using these data, energy consumption was recorded as kcal/cage. Body weight was measured weekly, and body composition was measured with EchoMRI™ 1100 Analyzer (EchoMRI LLC, USA) at baseline and on intervention weeks 6 and 12. At the end of the 12-week dietary intervention, 20-week-old rats were sacrificed under terminal anesthesia. Power calculations (n = 15 animals/group) were based on our earlier pilot experiment in which the effect of a Western diet on marrow adiposity was evaluated with histology, as described in the section Histology (power 90%; α = 0.05). The use of animals for experimentation was approved by the State Provincial Office of Southern Finland (ESAVI/30584/2022) and followed the Institutional Animal Care guidelines.

### Collection of tissues

Samples (100-200 mg) of perigonadal WAT and interscapular BAT were dissected and snap-frozen in liquid nitrogen. BMAT was isolated from the marrow cavity of the femur and tibia. In brief, metaphyseal ends of long bones were cut, and bones were centrifuged at 1000 × *g* for 30 seconds to evacuate bone marrow content into a collection tube containing 1 mL Hanks balanced salt solution (Gibco, USA; supplemented with 10 mM HEPES, 2% BSA, 50 U/mL heparin, 1 mM EDTA, 1 g/L L-glucose, and 0.25% sodium citrate). The bottom layer of stromal-vascular fraction (SVF), mainly devoid of adipocytes, was collected for purity analysis. The top floating layer of BMAT-enriched fraction was next transferred onto a 10 µm cell strainer (PluriSelect, Germany) and centrifuged at 400 × *g* at 4 °C for 10 minutes to remove residual bone marrow cells that were loosely attached to BMAT. SVF and BMAT-enriched samples were snap-frozen, and all samples were stored at −80 °C. The use of collagenase digestion to obtain purer samples of BMAds was omitted, as recommended by recent guidelines from the International Bone Marrow Adiposity Society (26). The presence of adipocytes in BMAT-enriched samples was confirmed by staining for neutral lipids using 5 µg/mL BODIPY 493/503 (Invitrogen, USA) and for nuclei using 5 µg/mL Hoechst 33258 (Invitrogen, USA) and imaged with an EVOS M5000 Imaging system (Thermo Fischer Scientific, USA). In addition, intact humerus and samples of WAT, BAT, and liver were collected for histology.

### Total RNA extraction and gene expression analysis

Adipose tissue samples (WAT and BAT) were first homogenized with UltraTurrax T25 (Janke and Kunkel IKA-Labortechnik, Germany) in 5-second bursts at 9500 rpm on ice. The oil layer released from lysed adipocytes was discarded. Next, total RNA was extracted with TriSure (Bioline, UK) according to the manufacturer's protocol. For BMAT samples, RNA isolation with TriSure was followed by an additional column-based purification using RNeasy Mini Kit (QIAGEN, Germany) according to the manufacturer's protocol. RNA yield was evaluated with NanoDrop One C (Thermo Fisher, USA), and the RNA integrity number was obtained with a 2100 Bioanalyzer (Agilent, USA). Total RNA (1 µg) was reverse transcribed to cDNA in a reaction mixture of Oligo(dT) (New England Biolabs, USA), deoxyribonucleoside triphosphates (Thermo Fisher, USA), RNase inhibitor (Promega, USA), and Maxima Reverse Transcriptase (Thermo Fisher, USA) with Mastercycler 5331 gradient (Eppendorf, USA).

Gene expression analysis with qPCR was performed with a Bio-Rad CFX96 thermal cycler (Bio-Rad, USA) using 100 ng cDNA, 25 µM primers, and a Dynamo SYBR Green HS qPCR kit (Thermo Fisher, USA) according to the manufacturer's protocol. Each primer sequence and target mRNA are listed in Table S1 (27). Data were analyzed by the Livak method (28) after normalizing each mRNA expression to peptidylprolyl isomerase B (*Ppib*).

### RNA-seq and transcriptomic analyses

A subset of 5 or 6 samples of WAT, BAT, and BMAT was selected from both intervention groups (n = 15/group) for RNA-seq. As the mRNA expression of *Fabp4* has been shown to be upregulated in bone marrow by a high-fat diet (29-31), the selection criteria included the response of BMAT to the diet as evaluated by mRNA expression of *Fabp4* by qPCR relative to the chow-fed group (+47%; *P* < .05) and an acceptable RNA integrity number. Samples of WAT and BAT from the same animals were selected. mRNA library preparation, RNA-seq, and bioinformatic analyses were purchased from Novogene GmbH (Munich, Germany). Briefly, the mRNA library was prepared with poly-A enrichment. Samples were then sequenced using Illumina NovaSeq 6000 with paired-end 150 bp strategy, and 20 M reads per sample. For read alignment, the HISAT2 algorithm was used to map the reads to the *Rattus norvegicus* reference genome (mRatBN7.2). Variation in the transcript profile between samples and study groups was analyzed with principal component analysis (PCA). Differentially expressed genes (DEGs) were identified with the DESeq2 algorithm and shown visually as a volcano plot or heatmap by transforming the data as log2-fold change or row *z* score, respectively, on transcripts with *P* < .05. The list of DEGs shown in each heatmap was sorted based on log2-fold change.

Next, we performed pathway-level gene-set enrichment analysis (GSEA; RRID: SCR_003199) with GSEA version 4.4 (32). Transcriptomic data were sorted by the signal-to-noise ratio metric and analyzed based on gene sets from Gene Ontology (GO) terms. In rodents, adipocytes represent less than 50% of the whole cell population in WAT, as sorted by recoverable nuclei in single-nucleus RNA-seq–based studies (33-35). Given previous reports on the differences in lipid metabolism between BMAT and WAT (14), we focused on the top 10 pathways related to lipid metabolism, by including in the analysis pathway names that contained the following keywords: “adipo-”, “lipid”, “sterol” and “fatty acid”. For GSEA statistics, enriched pathways with a false discovery rate (FDR) < 0.25 were considered statistically significant and set for further investigation of that gene set. A representative top-level pathway was chosen based on the occurrence of similar pathways from the top 10 pathways related to lipid metabolism. Finally, selected transcripts from that representative pathway were verified with qPCR, as previously described, in BMAT and WAT from the whole cohort.

### Histology

Samples of WAT, BAT, and liver as well as intact humerus were fixed in 10% neutral-buffered formalin for 48 h. The humerus was decalcified with 10% EDTA, 1 M NaH_2_PO_4_, and 0.5 M Na_2_HPO_4_ for 16 days. Samples were paraffin-embedded, sectioned as 4 µm histological sections, stained with hematoxylin and eosin, and scanned with Pannoramic P1000 (3DHistech, Hungary). Individual adipocyte area was semiautomatically measured using ImageJ (RRID: SCR_003070) from a single 1 mm × 1 mm region in WAT, annotated randomly and, in humeral bone marrow sections, annotated 5 mm distal from the proximal growth plate, as previously described (36). Briefly, the script targets the delipidated region of adipocytes between 200 µm^2^ and 4000 µm^2^ for BMAds or between 300 µm^2^ and 9000 µm^2^ for white adipocytes (WAds). Individual adipocyte size distribution was plotted according to bin widths of 200 µm^2^ and 600 µm^2^ for BMAds and WAds, respectively. The accumulation of lipid droplets in hepatocytes was quantitated from the whole slide images of hematoxylin and eosin–stained histological liver sections with a deep learning-based image analysis model utilizing Aiforia Create cloud platform version 5.3 (Aiforia technologies, Finland; RRID: SCR_022739), as previously described (37). Briefly, the area for microvesicular or macrovesicular steatosis was based on the detection of lipid droplets with a diameter less than or more than 6 µm, respectively, within the hepatocytes.

### Blood samples

Blood samples were collected into lithium heparin-containing tubes with a cardiac puncture at the time of sacrifice. Blood samples were immediately centrifuged at 200 × *g* for 10 min; plasma samples were obtained and stored at −80 °C until analysis. Plasma glucose levels were measured using Glucose Assay Kit (STA-681, Cell Biolabs Inc, USA) and total and free cholesterol were measured with the Cholesterol Quantification Assay Kit (CS0005, Sigma-Aldrich, Germany) according to the manufacturers' instructions. Measurements were performed with EnSight multimode plate reader (Revvity Oy, Finland).

### Bone microarchitecture and bone mineral density

Humeral length was measured with a caliper. The humerus was scanned with µCT SkyScan 1272 (Bruker, USA) at 70 kV, 142 µA, and a spatial voxel resolution of 10 µm. The growth plate at the proximal humeral metaphysis was located as a reference point for the analysis of the trabecular region, which spanned 2 mm distally. Next, the most distal region of the deltoid tuberosity was selected as a reference point for the analysis of the cortical region, which spanned 1 mm distally. Bone mineral density was measured by referencing the attenuation coefficient of each region to that of phantom materials with known density.

### Statistical analysis

Data are presented as box plots that represent the median value with interquartile range and whiskers that represent minimum and maximum values, whenever applicable. Each dataset was first tested for normal distribution with the Shapiro-Wilk normality test. Groupwise mean comparison was performed with a Student *t* test for normally distributed data or Mann-Whitney *U* test for non-normally distributed data. *P* values < .05 were considered statistically significant. Correlation coefficient R was computed with Pearson or Spearman correlation test for normally or non-normally distributed data, respectively. For qPCR analysis of adipose tissue comparison, 1-way ANOVA with Dunnett's correction for multiple comparisons was performed. For adipocyte size distribution analysis, 2-way ANOVA with Sidak's correction for multiple comparisons was performed. Adjusted *P* values < .05 were considered statistically significant. To reduce bias, data collection was performed using unique identifiers blinded to the experimental group.

For RNA-seq data, *P* values and adjusted *P* values or FDR were computed in DESeq2 and GSEA software. Adjusted *P* values (*P*_adj_) < .05 or FDR < 0.25 were considered statistically significant. Statistical analyses and graphing were performed in GraphPad Prism 10.6.1 (Graphpad Software LLC, USA).

## Results

### Western diet results in metabolic and skeletal changes in rats

**Figure 1:**
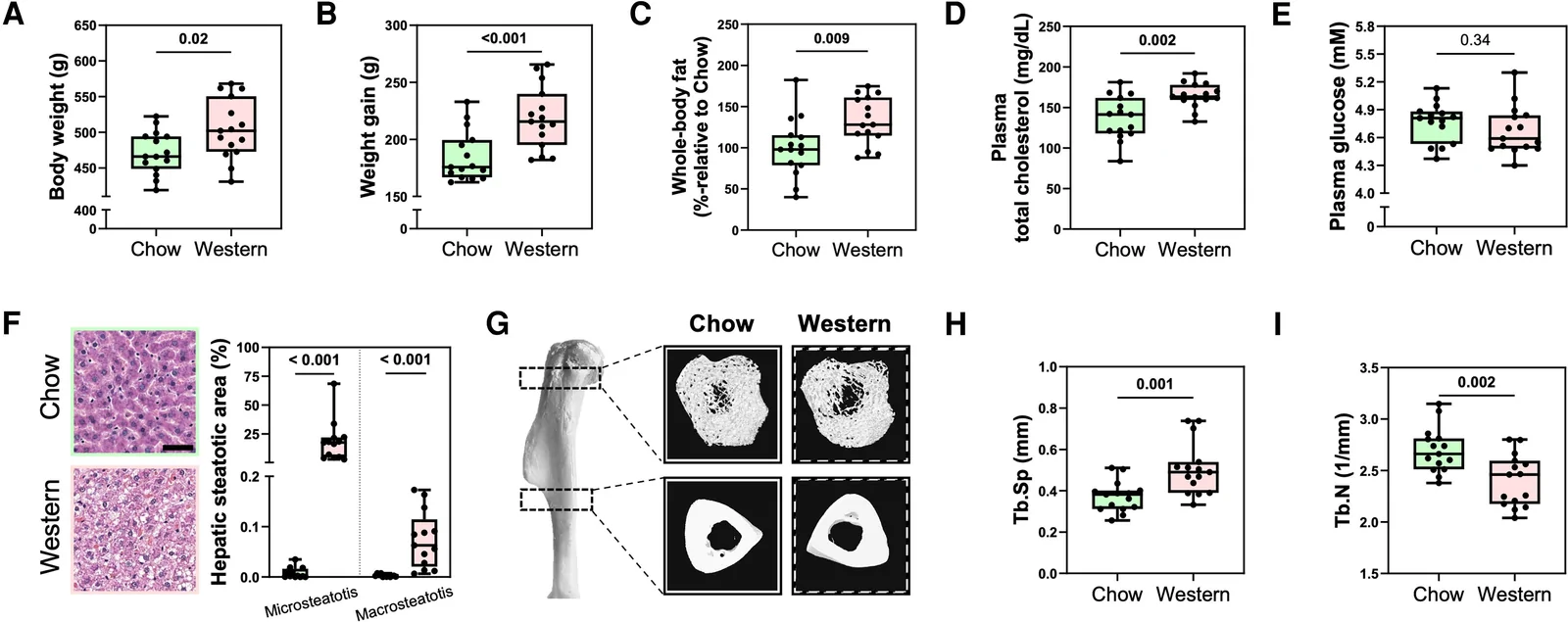
Phenotypical changes associated with a 12-week Western diet. Analyses of (A) body weight, (B) body weight gain, (C) relative change in whole-body fat fraction, (D) nonfasting plasma total cholesterol, and (E) nonfasting plasma glucose (all n = 15/group). (F) Histological analysis of steatotic lesions from whole sections of formalin-fixed, paraffin-embedded liver samples (n = 12-13/group). Scale bar: 50 μm. (G) Representative 3D renders of humerus microarchitecture evaluated by micro-computed tomography and the analyses of trabecular bone microarchitecture parameters (H) trabecular separation (Tb.Sp) and (I) trabecular number (Tb.N; all n = 15/group).

Rats with the 12-week dietary intervention had significantly higher body weight (mean, +7.1%; *P* = .02) (Fig. 1A), weight gain (+18.1%; *P* < .001) (Fig. 1B), and whole-body fat percentage (+33.2%; *P* = .009) (Fig. 1C) compared with the chow group. On average, rats fed a Western diet had persistently lower food consumption by weight than those fed a chow diet, despite having higher energy intake when adjusted for metabolizable energy of each diet component (*P* < .05; data not shown). Total plasma cholesterol was significantly higher in the Western diet group than in the chow group (+20.4%, *P* = .002) (Fig. 1D), but there was no difference in plasma glucose levels (−1.76%, *P* = .34) (Fig. 1E). We found significantly larger areas of microsteatotic and macrosteatotic lesions in the liver samples of the Western diet group compared with the chow group (both *P*s < .001) (Fig. 1F). Further, we observed changes in humeral trabecular region (Fig. 1G) as reflected by the significantly higher trabecular separation (+36.3%; *P* = .001) (Fig. 1H), lower trabecular number (−10.9%; *P* = .002) (Fig. 1I), and trabecular connectivity density (−20.5%; *P* = .009) (Table 1) in rats fed a Western diet. Bone mineral density in both trabecular and cortical regions was not significantly affected by the Western diet (all *P*s > .05) (Table 1).

**Table 1:** Humeral length, trabecular and cortical bone microarchitecture parameters, and bone mineral density analyses by micro-computed tomography, and correlation with the size of bone marrow adipocytes

Parameter	Chow diet	Western diet	R-coefficient vs BMAd.Ar	R-coefficient vs WAd.Ar
Bone length, mm	30.15 ± 0.23	30.16 ± 0.21	0.455	0.018
Tb.BV/TV, %	23.63 ± 4.10	21.39 ± 2.34	**−0**.**480**^a^	−0.047
Tb.N, 1/mm	2.70 ± 0.22	**2.41** **±** **0.25**^b^	**−0**.**673**^b^	−0.124
Tb.Th, mm	0.087 ± 0.01	0.089 ± 0.59	0.013	0.072
Tb.Sp, mm	0.372 ± 0.07	**0.50** **±** **0.13**^b^	0.288	0.164
Conn.D, 1/mm^3^	174.79 ± 36.39	**138.93** **±** **26.01**^b^	**−0**.**666**^b^	−0.118
Tb.BMD, g/cm^3^	0.95 ± 0.08	0.90 ± 0.12	0.032	−0.200
Ct.Th, mm	0.352 ± 0.13	0.435 ± 0.16	0.282	0.246
Ct.Po, %	2.74 ± 1.69	2.30 ± 1.66	−0.116	−0.214
Ct.Ar/Tt.Ar, %	97.26 ± 1.69	97.70 ± 1.66	0.116	0.214
Ps.Pm, mm	19.98 ± 3.75	18.28 ± 2.71	−0.073	−0.029
Es.Pm, mm	14.76 ± 0.62	15.06 ± 0.61	0.061	0.378
Ct.BMD, g/cm^3^	1.18 ± 0.11	1.16 ± 0.09	0.287	−0.056

Data are represented as mean ± SD. Groupwise comparison of each parameter was calculated by Student *t* test or Mann-Whitney *U* test based on the Shapiro-Wilk normality test for data distribution. Correlation coefficient was calculated by Pearson's or Spearman's correlation based on the Shapiro-Wilk normality test for data distribution.

Abbreviations: BMAd.Ar, Area of bone marrow adipocyte; Conn.D, connectivity density; Ct.Ar/Tt.Ar, cortical area per tissue area; Ct.BMD, cortical bone mineral density; Ct.Po, cortical porosity; Ct.Th, cortical thickness; Es.Pm, endosteal perimeter; Ps.Pm, periosteal perimeter; Tb.BMD, trabecular bone mineral density; Tb.BV/TV, trabecular bone volume per tissue volume; Tb.N, trabecular number; Tb.Sp, trabecular separation; Tb.Th, trabecular thickness; WAd.Ar, area of white adipocyte.

^a^ *P* < .05

^b^ *P* < .01

### BMAT is distinct from WAT and BAT and is enriched in pathways and transcripts related to the proliferation of adipoprogenitor cells

**Figure 2:**
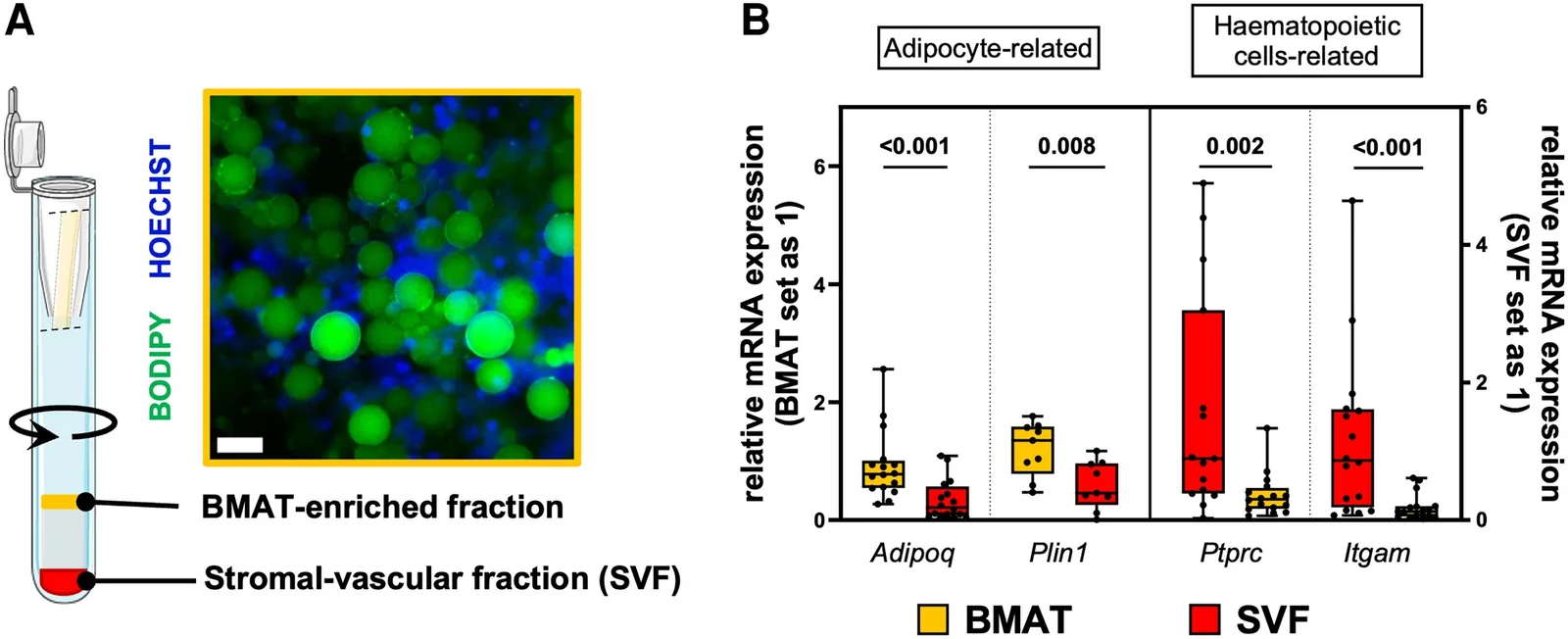
Collection of bone marrow adipose tissue (BMAT)-enriched fraction from the tibia and femur. (A) Schematic overview of the separation of stromal-vascular fraction and floating BMAT-enriched fraction by centrifugation, and the presence of bone marrow adipocytes confirmed by staining for neutral lipid and nucleus. Scale bar: 50 μm. (B) Analysis of sample purity by quantitative PCR revealed significantly higher mRNA expression for adipocyte-related markers *Adipoq* and *Plin1*, and significantly lower for hematopoietic-related markers *Ptprc* and *Itgam* in BMAT relative to the stromal-vascular fraction (n = 21). Illustration in (A) is provided by Servier Medical Art (https://smart.servier.com), licensed under CC BY 4.0 (https://creativecommons.org/licenses/by/4.0/).

**Figure 3:**
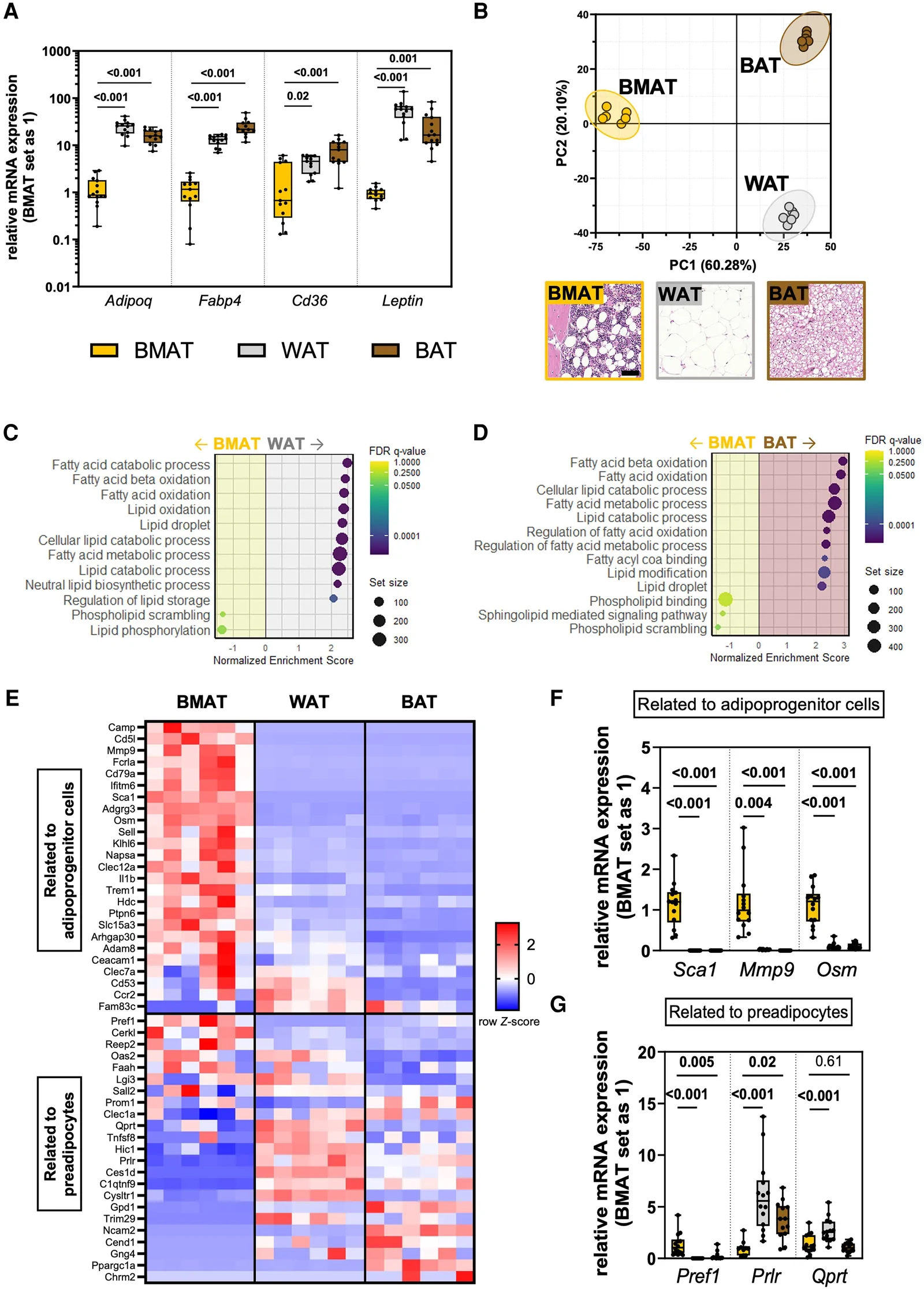
Bone marrow adipose tissue (BMAT) is a distinct fat depot enriched in transcripts associated with APCs. (A) BMAT expressed significantly lower adipocyte-related mRNA *Adipoq, Fabp4, Cd36*, and *Leptin* in comparison to white and brown adipose tissue (WAT and BAT, respectively) under basal conditions without dietary challenge (n = 15/tissue). (B) PCA of BMAT, WAT, and BAT transcriptomes, and their representative histological images. Scale bar: 75 μm. Targeted gene-set enrichment analysis focusing on pathways related to lipid metabolism in (C) BMAT vs WAT and (D) BMAT vs BAT revealed enrichment in pathways related to membrane lipid processes. (E) Analysis of differentially expressed genes (DEGs) related to adipoprogenitor cells (upper panel) and preadipocytes (lower panel). Selected gene sets are based on the dataset generated from Ambrosi et al (20). Verification of select DEGs with quantitative PCR from the whole cohort (n = 14/tissue) on transcripts related to (F) adipoprogenitor cells (eg, *Sca1, Mmp9*, and *Osm*), and (G) preadipocytes (eg, *Pref1, Prlr*, and *Qprt*).

First, we compared transcriptomes of 3 adipose depots in chow-fed rats (ie, without dietary challenge). The collected BMAT-enriched fractions were positive for neutral lipid and nuclear staining (Fig. 2A) and expressed significantly more mRNA for adipocyte-related markers perilipin-1 (*Plin1*) and *Adipoq,* and they expressed significantly fewer hematopoietic-related markers integrin alpha M (*Itgam*) and protein tyrosine phosphatase receptor (*Ptprc*) compared with SVF (Fig. 2B), highlighting the enrichment of adipocytes in BMAT samples. BMAT expressed significantly less mRNA for adipocyte-related markers *Adipoq, Fabp4,* cluster of differentiation 36 (*Cd36*), and *Leptin* compared with WAT and BAT (all *P*s < .05) (Fig. 3A). PCA of the entire RNA-seq data revealed 3 separate clusters representing BMAT, WAT, and BAT transcriptomes (Fig. 3B). In BMAT, GSEA revealed the top 10 enriched pathways related to biological processes that are tied to cellular proliferation (eg, spliceosome- and chromatin-related complexes) (Fig. S1A and S1B) (27). In WAT, pathways related to lipid metabolism (eg, GO terms fatty acid beta-oxidation and fatty acid catabolism) and extracellular matrix (eg, extracellular matrix organization, basement membrane and collagen trimer) were enriched (Fig. S1A), while those related to oxidative phosphorylation and mitochondrial energy metabolism were enriched in BAT (Fig. S1B). To highlight pathways that were enriched by the contribution of adipocytes within each adipose tissue sample, we further filtered the results using the predefined pathway set related to lipid metabolism, as described in detail in the previous discussion. In BMAT, pathways related to membrane-lipid processes (eg, phospholipid scrambling and lipid phosphorylation; all FDRs < 0.25) were more enriched when compared with WAT (Fig. 3C) and BAT (Fig. 3D). In WAT and BAT, pathways related to adipocyte physiology (eg, fatty acid beta oxidation, fatty acid metabolic process, and lipid droplet; all FDRs < 0.25) were more enriched than in BMAT (Fig. 3C and 3D).

The top enrichments in BMAT that were related to cell proliferation and membrane-lipid processes led us to further investigate transcripts related to less-differentiated adipocyte populations in BMAT. Using previously published lists of transcripts (20) known to be enriched in the APCs and preadipocyte population, we found in BMAT an abundance of transcripts related to the APCs, but not preadipocytes, when compared to WAT and BAT (Fig. 3E). qPCR analysis of the entire chow-fed cohort (n = 15) confirmed that the transcripts related to APCs (eg, stem cell antigen-1 [*Sca1*], matrix metalloproteinase-9 [*Mmp9*], and oncostatin M [*Osm*]) were expressed at significantly higher levels in BMAT when compared with WAT and BAT (all *P*s < .01) (Fig. 3F). In contrast, transcripts related to more mature preadipocytes (eg, prolactin receptor [*Prlr*], and quinolinate phosphoribosyltransferase [*Qprt*]) were expressed at lower levels in BMAT than in WAT and BAT (Fig. 3G).

### Western diet induces hypertrophy in both BMAds and WAds

**Figure 4:**
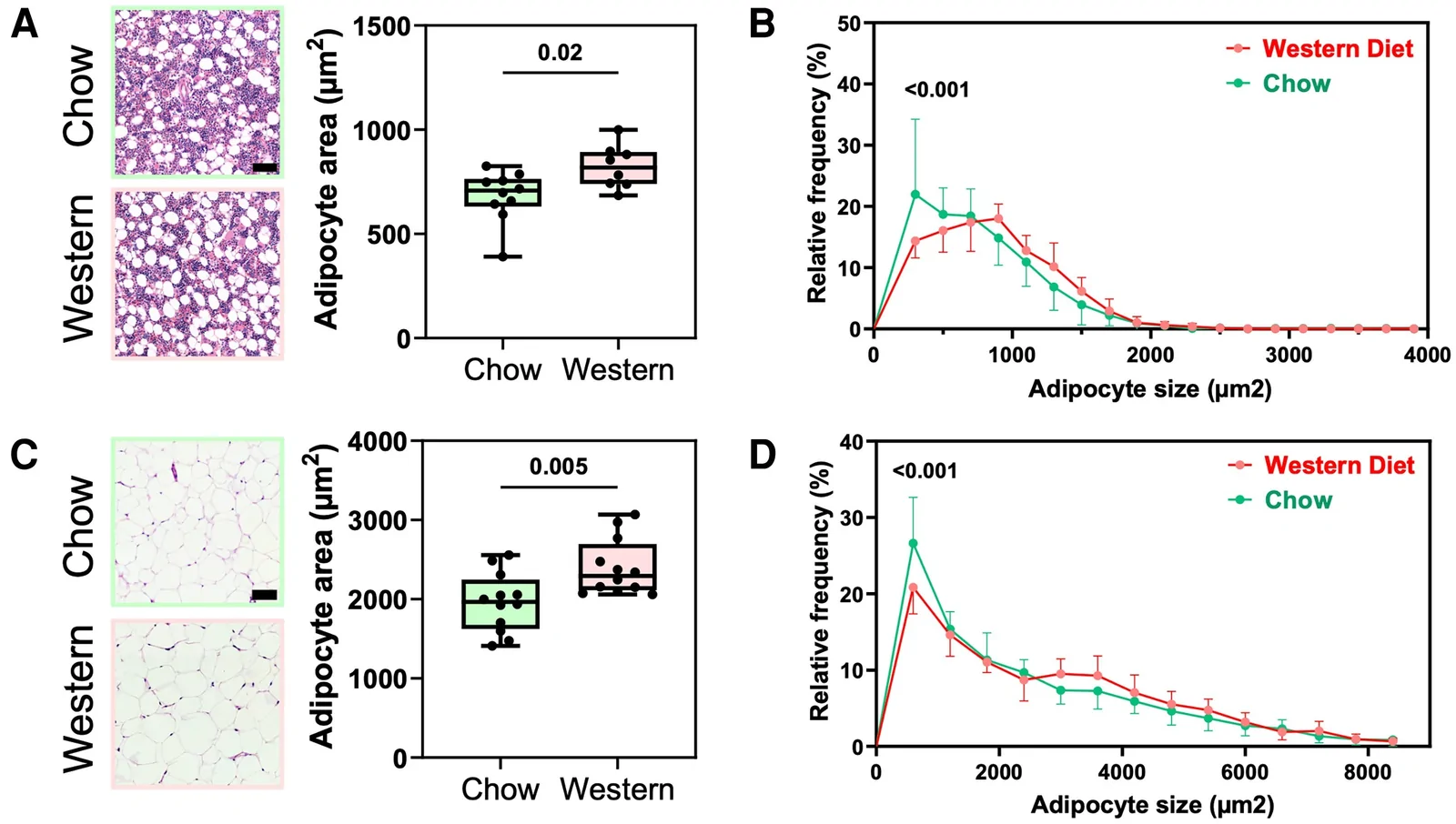
A 12-week Western diet significantly increases the size of bone marrow adipocytes (BMAds) and white adipocytes (WAds). (A) Representative images of humeral bone marrow histological sections, and the average size of BMAds (n = 8-10/group). (B) Histogram analysis of the frequency of BMAds, bin width 200 μm^2^ between bin centers of 300 μm^2^ and 3900 μm^2^. (C) Representative images of gonadal white adipose tissue (WAT) histological sections, and the average size of WAds (n = 12/group). (D) Histogram analysis of the frequency of WAds, bin width 600 μm^2^ between bin centers of 600 μm^2^ and 8400 μm^2^. Scale bars: 75 μm.

Next, we evaluated changes in the morphology of adipocytes in response to the 12-week Western diet intervention. On average, BMAds were smaller than WAds. Histological analysis of individual adipocyte size revealed that the Western diet significantly increased the size of BMAds (averaged 822 µm^2^ vs 681 µm^2^; +20.7%; *P* = .02) (Fig. 4A) in the proximal bone marrow of the humerus. The average number of BMAds in the Western diet group (292 BMAds per region of interest) was similar to that in the chow group (277 BMAds; *P* = .70). In line with this, the frequency of small BMAds, particularly those sized <400 µm^2^, was significantly lower in the Western diet group compared with the chow group (*P* < .001; Fig. 4B). Similarly, Western diet significantly increased the size of WAds in WAT (2399 µm^2^ vs 1958 µm^2^, +22.5%; *P* = .005) (Fig. 4C). The frequency of small adipocytes sized <900 µm^2^ was significantly lower in rats fed a Western diet (*P* < .001) (Fig. 4D). In addition, the size of BMAds, but not WAds, was inversely correlated with trabecular bone volume fraction (R = −0.48, *P* = .04) and trabecular number (R = −0.67; *P* = .002) (Table 1).

### The hypertrophic response of BMAds to a Western diet is associated with enrichment in pathways related to lipid transport

**Figure 5:**
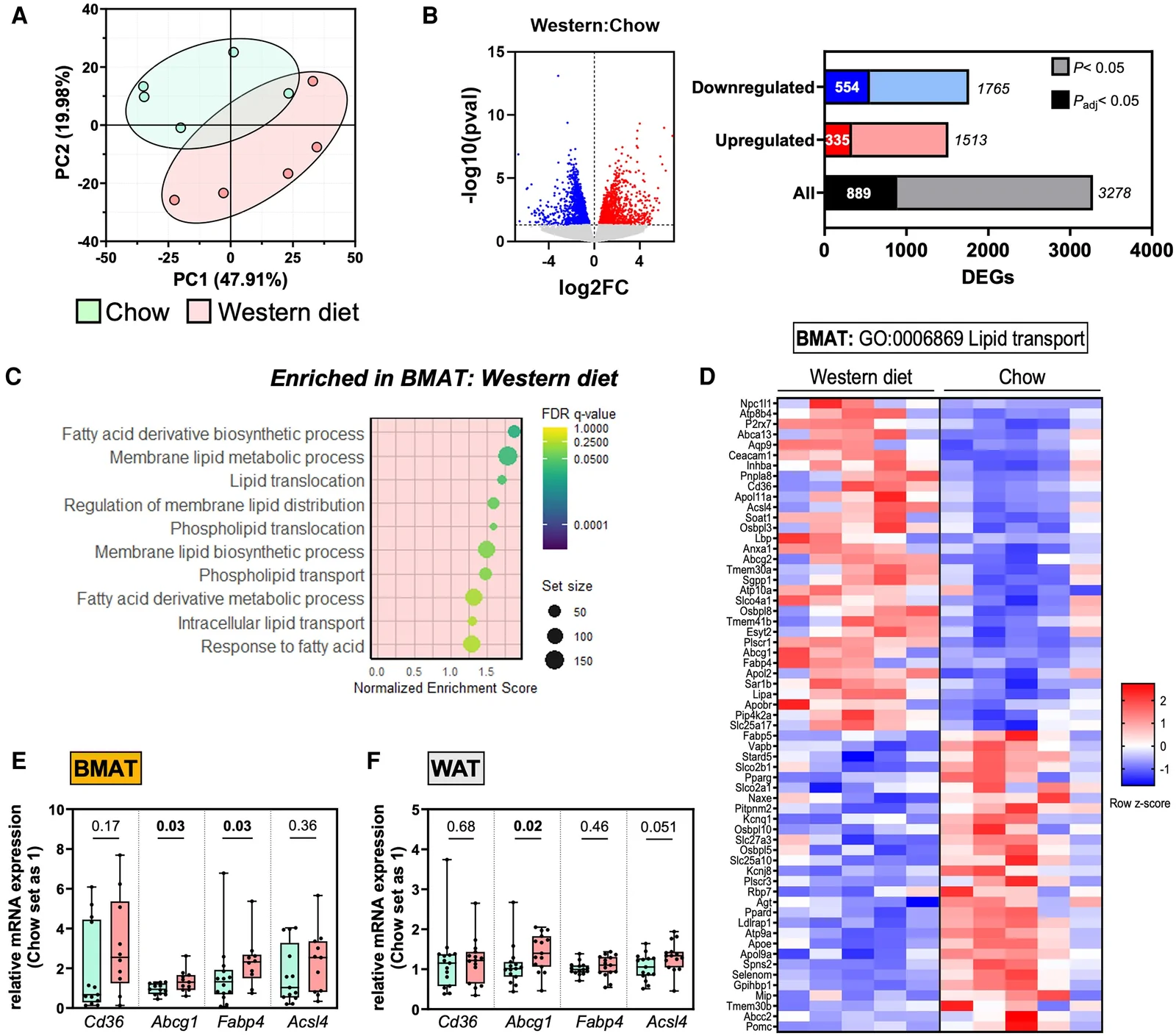
The expansion of bone marrow adipocytes (BMAds) upon a Western diet is associated with enrichment in pathways related to lipid transport. (A) PCA of bone marrow adipose tissue (BMAT) revealed 2 separate clusters representing BMAT transcriptomes in the 2 study groups. (B) Transcriptomic analysis revealed 889 differentially expressed genes (DEGs) (adjusted *P* < .05) consisting of 335 upregulated and 554 downregulated DEGs. (C) Targeted gene set enrichment analysis (GSEA) on pathways related to lipid metabolism revealed overall enrichment in several pathways related to lipid transport in the Western diet group (all false discovery rates < 0.25). (D) Transcript analysis on those transcripts annotated in the gene ontology-based lipid transport pathway (GO:0006869; all *P*s < .05) in BMAT in control and Western diet groups. Verification of selected DEGs with quantitative PCR from the whole cohort (n = 10-15/group) on transcripts related to lipid transport, *eg*, *Cd36*, *Abcg1, Fabp4* and *Acsl4,* in (E) BMAT and (F) white adipose tissue (WAT).

To understand the molecular mechanism driving the hypertrophy of BMAds on a Western diet, we compared BMAT transcriptomes in rats fed with either chow or a Western diet. PCA revealed 2 separate clusters of transcriptomes, corresponding to the chow and Western diet groups (Fig. 5A). Further, we identified 3278 DEGs (n = 889 after statistical adjustment), of which 1513 (46%) were upregulated (n = 335; 38%) and 1765 (54%) were downregulated (n = 554; 62%) (Fig. 5B). In GSEA, we first focused on pathways related to lipid metabolism. The fatty acid derivative biosynthetic process (accession number GO:1901570; FDR = 0.01) was the top enriched pathway in BMAT in response to the Western diet. Consistently, several pathways related to lipid transport appeared to be enriched with a Western diet, such as lipid translocation (GO:0034204; FDR = 0.04), regulation of membrane lipid distribution (GO:0097035; FDR = 0.07), phospholipid transport (GO:0015914; FDR = 0.12), and intracellular lipid transport (GO:0032365; FDR = 0.25) (Fig. 5C). Based on the enrichment related mainly to the lipid transport, we discovered 4 core-enriched transcripts annotated in the lipid transport pathway (GO:0006869), including *Cd36,* ATP-binding cassette subfamily G member 1 (*Abcg1*)*, Fabp4*, and acyl-coenzyme A synthetase long-chain family member 4 (*Acsl4*). Using qPCR analysis from the entire animal cohort, we verified in BMAT a significant upregulation in the expression levels of *Abcg1* (+32%; *P* = .03) and *Fabp4* (+47%; *P* = .03) and an increasing, but not significant, trend for the expression of *Cd36* (+63%; *P* = .17) and *Acsl4* (+44%; *P* = .36) in response to a Western diet (Fig. 5E). In WAT, Western diet did not significantly affect these selected transcripts (all *P*s > .05) related to lipid transport, except for *Abcg1* (+31%; *P* = .02) (Fig. 5F).

**Figure 6:**
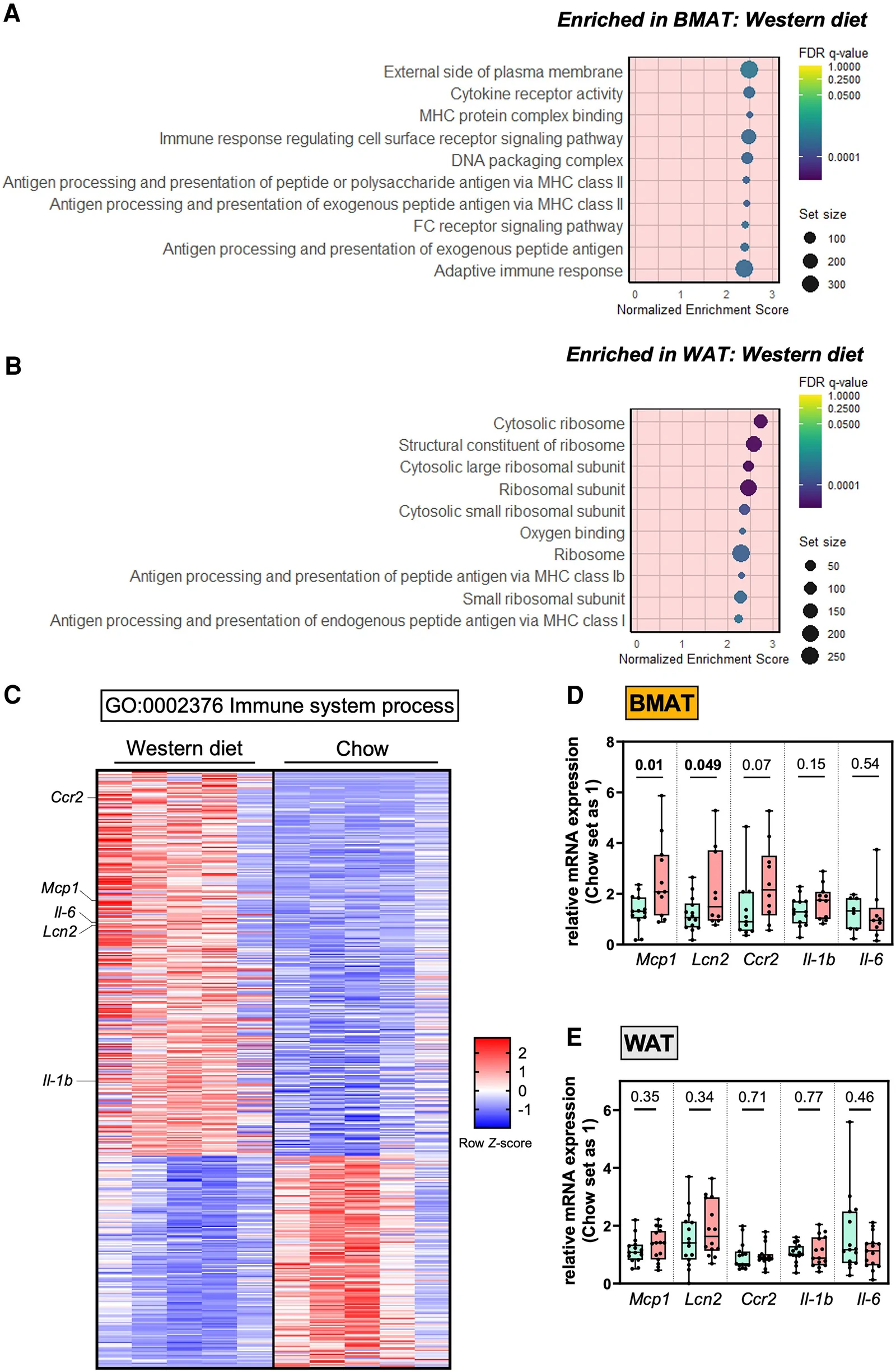
The transcriptomes of bone marrow adipose tissue (BMAT), but not white adipose tissue (WAT), are enriched in pathways related to the inflammation process upon Western diet. Top 10 unfiltered pathways from gene set enrichment analysis (GSEA) between respective study groups in (A) BMAT and (B) WAT. (C) Transcript analysis on those transcripts annotated in the gene ontology-based immune system process pathway (GO:0002376; all *P*s < .05). Verification of selected DEGs with quantitative PCR from the whole cohort (n = 10-15/group) on transcripts related to adipocyte-associated immune system process (eg, *Mcp1, Lcn2, Ccr2, Il1b*, and *Il6*) in (D) BMAT and (E) WAT.

We then evaluated GSEA on a global level without data filtering (Fig. 6A and 6B). In BMAT, the top 10 GSEA enriched by a Western diet revealed significant enrichments (all FDRs < 0.25) in pathways related to the immune system process (eg, cytokine receptor activity [GO:0004896] and antigen processing and presentation of exogenous peptide via MHC class II [GO:0019886]) (Fig. 6A). To better understand the putative inflammatory response in BMAT to a Western diet, we queried DEGs (all *P*s < .05) in our dataset, which are annotated in the GO database for the immune system process (GO:0002376). A Western diet significantly increased the expression of several transcripts related to adipose tissue inflammation (38), such as monocyte chemoattractant protein-1 (*Mcp1,* also known as *Ccl2*), Lipocalin-2 (*Lcn2*), C-C chemokine receptor type 2 (*Ccr2*), interleukin 1-β (*Il1b*) and interleukin-6 (*Il6*) (Fig. 6C). Upregulation of these transcripts was further verified in the entire cohort with qPCR, and we saw a significant upregulation of *Mcp1* (+114%; *P* = .01) and *Lcn2* (+81%; *P* = .05) in BMAT on a Western diet (Fig. 6D). Similar upregulation was not seen in WAT (Fig. 6E).

### The hypertrophic response of WAds to a Western diet is associated with the enrichment in pathways related to lipid biosynthesis and modification

**Figure 7:**
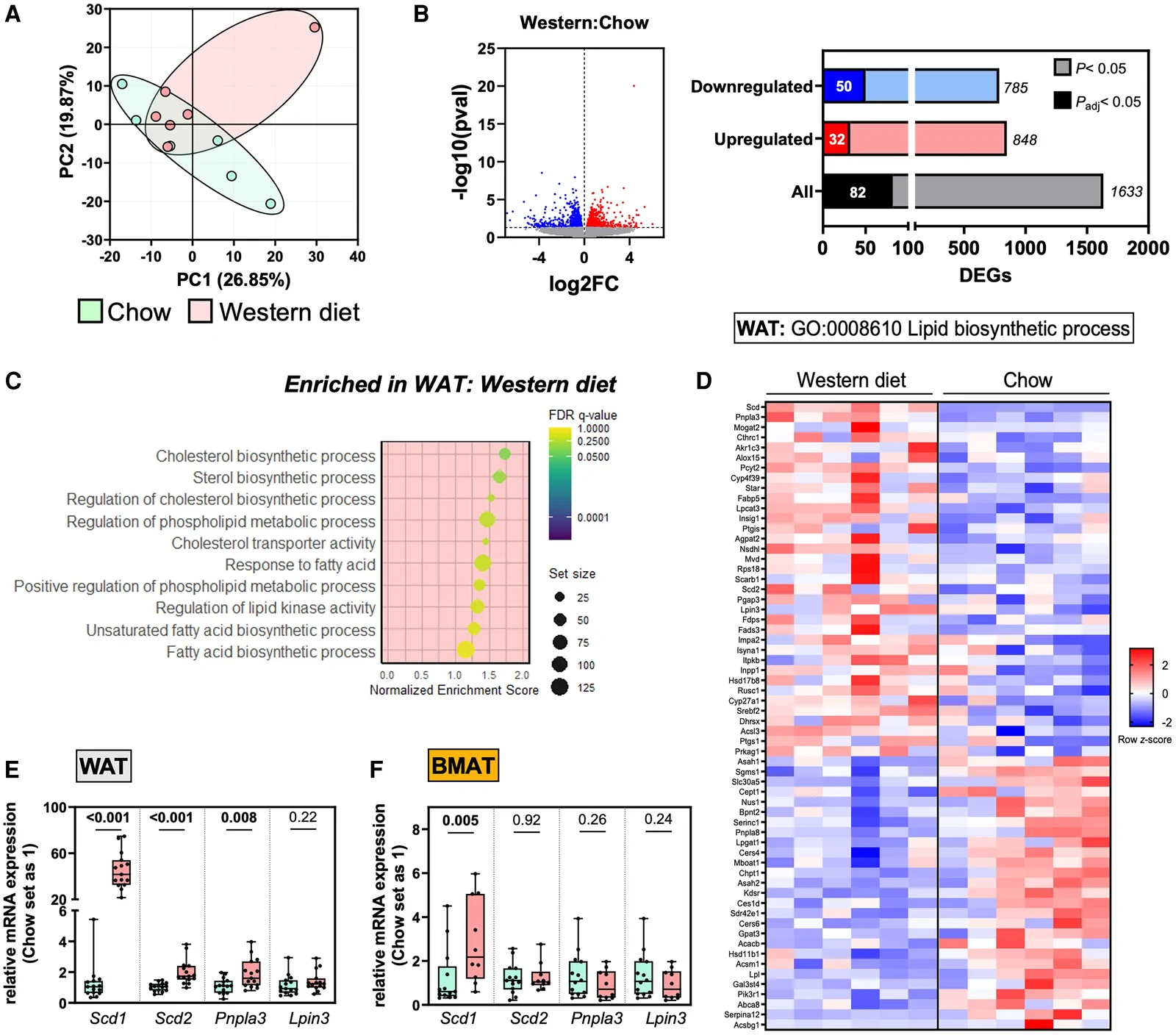
The expansion of white adipocytes (WAds) upon a Western diet is associated with enrichment in pathways related lipid biosynthesis and modification. (A) PCA of white adipose tissue (WAT) revealed 2 modestly separated clusters representing WAT transcriptomes in the 2 study groups. (B) Transcriptomic analysis revealed 82 differentially expressed genes (DEGs) (adjusted *P* < .05) consisting of 32 upregulated and 50 downregulated DEGs. (C) Targeted gene set enrichment analysis on pathways related to lipid metabolism revealed overall enrichment in pathways related to lipid biosynthesis and modification in the Western diet group (all false discovery rates < 0.25). (D) Transcript analysis on those annotated in the gene ontology-based lipid biosynthetic process pathway (GO:0008610; all *P*s < .05) in WAT in control and Western diet groups. Verification of selected DEGs with quantitative PCR from the whole cohort (n = 10-15/group) on transcripts related to the lipid biosynthetic process (eg, *Scd1, Scd2, Pnpla3*, and *Lpin3*) in (E) WAT and (F) bone marrow adipose tissue.

Similar to the analysis of BMAT, we performed bulk RNA-seq from samples of gonadal WAT in rats fed with either chow or a Western diet. PCA revealed a less pronounced separation in the transcriptome clusters of WAT between diet groups (Fig. 7A). We further found only 1633 DEGs (n = 82 after statistical adjustment), compared to 3278 DEGs in BMAT, of which 848 (52%) were upregulated (n = 32; 39%) and 785 (48%) were downregulated (n = 50; 61%) (Fig. 7B). The overall top 10 GSEA showed significant enrichments (all FDRs < 0.25) in pathways related to translation and protein synthesis (eg, cytosolic ribosome [GO:0022626] and ribosomal subunit [GO:0044391]) (Fig. 6B). When we focused the GSEA analysis on pathways related to lipid metabolism, we observed that several pathways related to lipid biosynthesis and modification, such as those involved in the cholesterol and fatty acid biosynthesis, were enriched in WAT with a Western diet (Fig. 7C). Based on the enrichments in lipid biosynthesis in WAT, we selected representative transcripts stearoyl-coenzyme A desaturase-1 and −2 (*Scd1* and *Scd2*, respectively), patatin-like phospholipase domain-containing protein 3 (*Pnpla3*), and lipin-3 (*Lpin3*) from the lipid biosynthetic process (GO:0008610) (Fig. 7D) and verified the expression in WAT and BMAT with qPCR. In WAT, we observed significant upregulation of *Scd1* (35-fold increase; *P* < .001), Scd2 (+93%; *P* < .001) and *Pnpla3* (+69%; *P* = .008) with a Western diet, but not *Lpin3* (Fig. 7E). In BMAT, we observed a modest but significant upregulation only for Scd1 (2.3-fold; *P* = .005) (Fig. 7F). Under normal conditions (ie, study group without a Western diet), the mRNA expression of *Scd1* was significantly lower in BMAT than WAT (12-fold; *P* < .001). There were also some similarities in the response of WAT and BMAT to a Western diet. For instance, the pathway entitled response to fatty acid (GO:0070542) was among the top 10 pathways related to lipid metabolism in both BMAT (Fig. 5C) and WAT (Fig. 7C) in response to a Western diet.

## Discussion

In this study, we compared the transcriptomes of 3 adipose tissue depots of BMAT, WAT, and BAT in rats and studied the effect of a high-fat, high-cholesterol Western diet on BMAT and WAT. Our data demonstrate that BMAT is transcriptionally different from WAT and BAT, and is enriched in transcripts related to the proliferation of less-mature APCs. BMAT and WAT show differential transcriptomic responses to a Western diet. The hypertrophy of BMAds is more related to the enrichment in pathways related to lipid transport, while the hypertrophy of WAds is more related to pathways involved in lipid biosynthesis and modification.

BMAT isolated from rat femur and tibia expressed adipocyte-related transcripts (eg, *Adipoq*, *Fabp4*, and *Leptin*) at significantly lower levels than WAT and BAT, similar to what others have observed in rodents (13, 24). On a pathway level, our results showed enrichment in pathways related to proliferation and phospholipid scrambling, which may suggest active cellular division. To identify the potential proliferating cell population, we further analyzed the transcriptome for the presence of APCs and preadipocytes that spatially associate with mature BMAds (16-18). Using curated lists of transcripts defining murine skeletal *Sca1*^+^ stem cell-like populations in bone marrow (20), we observed an abundance of several transcripts related to APCs. Among those, *Mmp9*, a matrix metalloproteinase, has been shown to be expressed abundantly in undifferentiated preadipocytes in rodents (20, 39) and humans (40, 41) and found to be inversely associated with adipogenic maturation (41). *Osm* was highly upregulated in our dataset, and it has been reported to inhibit adipogenesis progression in rodents (42). Collectively, our data may suggest that BMAT is a less mature adipose tissue than WAT and BAT, at least in young rats on a standard diet, or that BMAd progenitors are transcriptionally distinct from those for white and brown adipocytes. This is in line with studies reporting the continuous BMAT expansion during aging in rodents (5, 24, 43-45). The physiological significance of the relatively abundant presence of APCs in BMAT is presently unknown and therefore warrants more investigation.

We found that hypertrophy of BMAds and WAds in response to the dietary intervention occurs, at least in part, via different pathways. This is highlighted by the enrichment of different transcripts in these 2 different adipose tissue types. In BMAT, we observed enrichment in transcripts related to lipid transport. Bone marrow is a highly perfused organ in the hematopoietic-rich femoral red marrow, receiving higher perfusion than subcutaneous WAT (46). Further, we and others have earlier reported lipid and fatty acid uptake in adipocyte-rich bone marrow of long bones (7, 47, 48). Collectively, this may suggest a nutrient-sensing role for BMAT in clearing circulating lipids. Interestingly, our data did not show significant changes in transcripts for the classical adipocyte machinery for lipid uptake, including the fatty acid transport proteins (*Fatps*) family and lipoprotein lipase (*Lpl*), suggesting other mechanisms responsible for fatty acid uptake in BMAds. Bartelt and colleagues have shown increased fatty acid uptake in the bone marrow of adipocyte-specific *Lpl*^−/−^ mice, and they implied a lipoprotein lipase–independent hydrolysis of fatty acids for uptake in BMAds (47). We observed increases in the expression of *Cd36* and *Fabp4*, which are known to channel fatty acids into adipocytes and traffic them for storage. Notably, CD36 is known to channel up to 50% of fatty acids into 3T3-L1 adipocytes through caveolar endocytosis (49). Accordingly, our RNA-seq data show modest upregulation for machineries downstream of CD36-mediated fatty acid uptake, including Lck/Yes novel tyrosine kinase (*Lyn*), zinc finger DHHC-type palmitoyltransferase 5 (*Zdhhc5*), acyl-protein thioesterase 1 (*Lypla1*), and spleen-associated tyrosine kinase (*Syk*) (data not shown). Regardless, detailed investigation of the mechanisms of lipid uptake in BMAds remains to be comprehensively studied.

Our data show differential transcriptomic response between BMAT and WAT on a Western diet. In WAT, we observed enrichment in the transcripts related to lipid biosynthesis, including intracellular lipid processing and modification. In particular, *Scd1*, the rate-limiting enzyme responsible for the synthesis of monounsaturated fatty acids (50), was strikingly upregulated in WAT (35-fold) but only modestly in BMAT (2.3-fold). Such contrast further highlights the differential responses of BMAT and WAT to a Western diet. In *Scd1*^−/−^ mice, Ntambi and colleagues showed a marked reduction in triglyceride synthesis (51), thereby extending the role of *Scd1* beyond fatty acid desaturation and likely potentiating lipid storage and adipocyte hypertrophy in WAds. To clarify the term lipid biosynthesis, our data did not show enrichment in pathways related to carbohydrate handling in either WAT or BMAT, such as carbohydrate kinase activity (GO:0019200) and carbohydrate transmembrane transport (all FDRs > 0.25; data not shown), as adipocytes may accrue lipids through de novo lipogenesis from carbohydrate precursors. Further, we and others have shown suppressed glucose uptake in bone marrow and WAT in response to a high-fat diet (6, 52, 53), making de novo lipogenesis unlikely to explain lipid droplet growth apart from lipid uptake. Notably, we observed a similar enrichment in the GO term response to fatty acids (GO:0070542) in both BMAT and WAT in response to a Western diet, supporting the nutrient-sensing role of both adipose tissues. However, this is followed by a differential response of lipid transport in BMAT and lipid biosynthesis and processing in WAT.

Interestingly, our data show enrichment in pathways related to immune system processes in BMAT in response to a Western diet, which was not as pronounced in WAT. We further verified the significant upregulation of proinflammatory cytokines, such as *Mcp1* and *Lcn2*, in BMAT by Western diet. Our data are not in line with the previously reported noninflammatory phenotype of BMAT in mice on a 60 kcal% high-fat diet (13), suggesting perhaps a species-specific difference in the inflammatory response of BMAT on a high-fat diet. In humans, on the other hand, Fazeli and colleagues reported an increase in proinflammatory transcripts in mature primary BMAds on acute high-calorie feeding (54). Studies on changes related to the inflammation status tied to the expansion of BMAT on a Western diet in different species are therefore warranted.

We also observed significant inverse associations between the size of BMAds and trabecular bone microstructure, both assessed in humerus, which is in line with what we and others have reported in the tibia in rodents fed a high-fat diet (6, 13, 55, 56). However, the causal relationship between bone loss and bone marrow adiposity has yet to be determined, particularly as context-dependent bone loss may occur independently of BMAT expansion (57-59). Further, a supportive role of BMAds in bone formation has also been reported (10, 60). Future work is therefore needed to examine whether BMAds in the humerus follow the pattern of adipocyte heterogeneity in the tibia (61) and whether BMAd expansion alone or in combination with changes in the bone marrow environment on a Western diet potentiates trabecular bone loss.

The present study is not without limitations. We analyzed samples of BMAT from femur and tibia, WAT from the perigonadal region, and BAT from the interscapular region from male Sprague-Dawley rats at 20 weeks of age. Adipose tissues exhibit depot- and sex-specific and age-dependent characteristics (36, 43, 45, 62), thereby limiting interpretation of the data to other specific adipose depots or animals of different ages. Regardless, femoral and tibial BMAT represent 2 very well characterized subpopulations of BMAds, providing a holistic overview of the response of BMAT to a Western diet. Further, gonadal WAT used in this study has commonly been used as a surrogate for visceral fat depot that responds to obesogenic feeding, offering a practical and metabolically informative site for assessing adiposity in response to dietary intervention (63). Despite efforts to deplete SVF from our samples of BMAT, other nonadipocyte cells may have remained attached to BMAds and carried over to the analysis. We acknowledge that the estimation of cellularity of the samples (adipocytes vs other cell types) via, for example, cell sorting, was not evaluated in the present study, and it should be addressed in future studies. Further, the results of bulk RNA-seq data reflect changes captured at a tissue level; thereby, to some extent, our results may have been influenced by nonadipocyte cells in the samples. To address this, we employed a targeted analysis through stringent data filtering, which enabled us to focus specifically on pathways related to lipid metabolism. The omission of collagenase digestion, as recommended by the recent guidelines (26), enabled us to preserve the amount of BMAT and to perform RNA-seq on individual samples, which is not always feasible for the challenging isolation of BMAT from rodents (43, 64). The strengths of the study include global analysis of adipose tissue transcriptomes, which was further verified by qPCR on selected genes from the whole study cohort. In the future, the use of single-nucleus RNA-seq should enable the investigation up to cellular resolution, although the mRNA expression can also differ between nuclei and cytoplasm, further complicating the interpretation of the data (65). However, transcriptomic analyses alone cannot fully confirm differences in progenitor cells or metabolic functions. In addition, the use of a Western diet allowed us to improve the translatability and clinical relevance of the results, as a Western diet may mirror the gradual development of obesity in humans (66). The ongoing development of ex vivo cultures for primary BMAds (26) should allow us to further investigate the lipid uptake mechanism in BMAds in the future.

In summary, we present herein the global transcriptomic analysis of 3 adipose tissue depots of BMAT, WAT, and BAT. Our data suggest that transcripts related to less-mature APCs are overrepresented in BMAT, possibly reflecting the developmental maturity of BMAT. The expansion of BMAT involves, in part, pathways related to lipid transport, while the expansion of WAT may rely more on pathways involved in lipid biosynthesis and modification. Further, BMAT appears to be more responsive to the diet-induced transcriptomic changes than WAT. Collectively, the present study strengthens the concept of BMAT as a distinct adipose depot and provides new information on the enriched pathways in BMAT responding to dietary intervention.

## Data Availability

Original data generated and analyzed during this study are included in this published article or in the data repositories listed in References. The raw datasets generated and analyzed during this study are publicly available in Zenodo at https://doi.org/10.5281/zenodo.21023939
